# Free drug provision for tuberculosis increases patient follow-ups and successful treatment outcomes in the Indian private sector: a quasi experimental study using propensity score matching

**DOI:** 10.1186/s12879-023-08396-5

**Published:** 2023-06-21

**Authors:** Ridhima Sodhi, Michael J. Penkunas, Arnab Pal

**Affiliations:** 1 Clinton Health Access Initiative, Inc., New Delhi, India; 2 Clinton Health Access Initiative, Inc., Boston, United States

**Keywords:** Free drug provision, Tuberculosis, Private sector engagement, Propensity score matching; India

## Abstract

**Background:**

The private sector is an important yet underregulated component of the TB treatment infrastructure in India. The Joint Effort for Elimination of Tuberculosis (Project JEET) aims to link private sector TB care with the constellation of social support mechanisms available through the Indian National TB Elimination Programme (NTEP), including the provision of free fixed-dose combination (FDCs) drugs to patients. This quasi-experimental study analysed routinely collected data to determine the impact of free drugs on patient follow-ups and treatment outcomes.

**Methods:**

We used data for private sector patients enrolled with Project JEET who were diagnosed with pulmonary and extrapulmonary TB between 1 January 2019 and 31 March 2020, and completed treatment by 31 December 2021. Propensity score matching was used to create a dataset to compare the number of follow-ups and proportion of successful treatment outcomes for patients on free drugs to a control group who paid out-of-pocket. 11,621 matched pairs were included in the analysis. Logistic regression and ordinary least squares regression models were used to estimate the impact of free drugs on number of follow-ups and treatment success, where latter is defined as treatment completion or cure.

**Results:**

After controlling for potential confounders, patients on free drugs received on average 2.522 (95% C.I.: 2.325 to 2.719) additional follow-ups compared to patients who paid out of pocket. This equates to a 25% mean and 32% median increase in follow-ups for patients availing free drugs. For treatment success, patients receiving free drugs had 45% higher odds of a successful treatment (Odds Ratio: 1.452, 95% C.I.: 1.288 to 1.637).

**Conclusions:**

Patients receiving free drugs were found to follow up with their treatment coordinator more frequently, in part likely to enable drug refilling, compared to patients who were paying out of pocket. These additional contacts would have offered opportunities to address concerns regarding side effects, provide additional treatment information, and connect with social support services, all of which subsequently contributed to patients’ continual engagement with their treatment. This potentially represents the unmeasured effect of free drugs on continual social support, which translates into a higher odds of treatment success for patients.

**Supplementary Information:**

The online version contains supplementary material available at 10.1186/s12879-023-08396-5.

## Background

In 2021, an estimated 10.6 million people fell ill with tuberculosis (TB), an increase of 4.5% from 2020, after several years of decline [1]. Globally, TB is the leading cause of death due to infectious diseases, while being preventable and curable [2] [3]. India has the highest burden of TB in the world, accounting for more than 28% of the global TB incidence, and the highest number of TB deaths − 36% among HIV negative people and 32% among HIV positive people [1]. According to the latest WHO guidelines, treatment for pulmonary TB is spread across at least 6 months for drug susceptible TB patients, and is divided in two phases – 2 months of intensive phase (isoniazid, rifampin, pyrazinamide, and ethambutol) followed by a continuation phase of 4 months with isoniazid and rifampin [4]. It is typically longer in cases of extra pulmonary drug-susceptible cases. The long duration of treatment, along with the complicated drug regimen is a major barrier to adherence and has a significant negative impact on tuberculosis control [1, 5, 6].

With the death rate among non-treated being significantly high [7], universal health coverage for TB treatment is necessary, along with sustained efforts to ensure treatment adherence. The government of India offers free diagnosis and treatment for TB, for patients accessing care in the public sector [8]. However, more than half of the 2.6 million persons infected annually [9] access diagnostic and treatment services through the private health sector [10]. Although private sector providers in India offer several advantages to patients over the public sector [11], such as quicker appointment times, shorter distances to travel, or better service experience, significant gaps remain across TB the care cascade in the private sector. Additionally, private sector providers often lack TB-specific knowledge, use non-standardized treatment regimens, and patients face the potential for catastrophic healthcare costs [12–14]. Due to a general lack of medication adherence support, TB patients within the private sector face poorer treatment outcomes and an elevated risk of recurrent TB than those treated in the public sector [10, 15].

The Joint Effort for Elimination of Tuberculosis (Project JEET) is a large-scale private health sector engagement initiative for TB in India [16]. Running in close coordination with the National TB Elimination Programme (NTEP) [16], the program has run across 24 states and 488 districts, and is aimed at sensitising private sector healthcare providers on the latest TB guidelines and raising awareness about the significance of notifying TB patients [16]. Services offered through Project JEET are intended to mitigate challenges limiting the Indian health care system in arresting TB transmission, facilitating access to appropriate TB care, and supporting TB patients throughout their treatment – all with the intention of helping India meet its TB elimination goal by 2025. As part of the project, designated healthcare workers help support the notification of patients diagnosed with TB, thereby registering them in Nikshay – which is the government data management system for TB. The latter ensures that infected patients and their families can be provided with referrals and linkage to services provided by the NTEP, thus helping in limiting the onward transmission of the disease. Importantly, private providers engaged with Project JEET are sensitized to use free, quality assured diagnostic services (Xpert testing) for their patients and enable linkage of patients with a dedicated treatment coordinator allowing for free counselling services. Additionally, engaged providers are encouraged to make patients aware of the free government provided TB drugs in the form of fixed dosage combinations (FDCs) [16, 17]. While the majority of TB drugs prescribed in India are FDCs [18], they are often purchased out of pocket by patients. As part of Project JEET, designated treatment coordinators are responsible for enabling the availability of free drugs to the patients prescribed with them, and their refill monitoring, by coordinating with NTEP staff, private sector provider and the patient. We find that this additional task (over and above other tasks such as counselling, adherence support, and contact investigation) potentially adds to the number of contacts the treatment coordinator makes with the patient, likely providing patients with an additional opportunity to ask questions regarding their treatment. Our aim is to estimate the differential rates in patient follow-ups and successful TB treatment outcomes for patients who received free drugs compared to those who did not receive them.

## Methods

### Settings and Project JEET

This study assesses demographic and treatment related information of patients who sought treatment for TB through private sector facilities that were engaged with Project JEET, as a part of the Patent Provider Support Agency (PPSA) in India managed by the William J. Clinton Foundation (WJCF). PPSA is a model under which a third-party entity, such as a non-governmental organization, engages private sector TB physicians to provide end-to-end services for TB [19].

Patients treated under Project JEET were assigned a treatment coordinator who was responsible for regularly following up with the patient and counselling them through different stages of their treatment, either in-person or via telephone. In-person counselling typically took place at the treating facility or within patients’ homes. In some cases, patients preferred meeting the treatment coordinator at another place of their convenience.

Patients typically availed free drugs at the provider’s clinic itself, and these drugs are refilled every 28 days [20]. While the treatment coordinators were responsible for facilitating the process by liaising with the patients, the maintenance of dispensation records varied between geographies and changed over time. For instance, in Delhi, Gurgaon & Indore, the NTEP staff were often responsible for maintaining the data on drug re-fills for a majority of patients. This is relative to other districts where the JEET staff (treatment coordinators and hub agents) took primary responsibility. Additionally, these records were not always updated digitally, and were maintained in registers. Over the course of the project, the digital maintenance of drug refills became more widespread. In some cases, patients could shift to private drugs or vice-versa, depending on a change in provider’s prescription. For our analyses, a binary indicator is used to assess if a patient receives free drugs or not – depending on whether they have received at least one prescription (28 days) of free drugs or not. This information was recorded by the WJCF staff, to assess the percentage of patients receiving free drugs, and analyzing the providers using these services (or not).

### Study design

A quasi-experimental study, using a propensity score matched dataset of routinely collected programmatic data, for a retrospective cohort of patients, was conducted. Access to free drugs was the independent variable under investigation.

### Data source

**Fig. 1:**
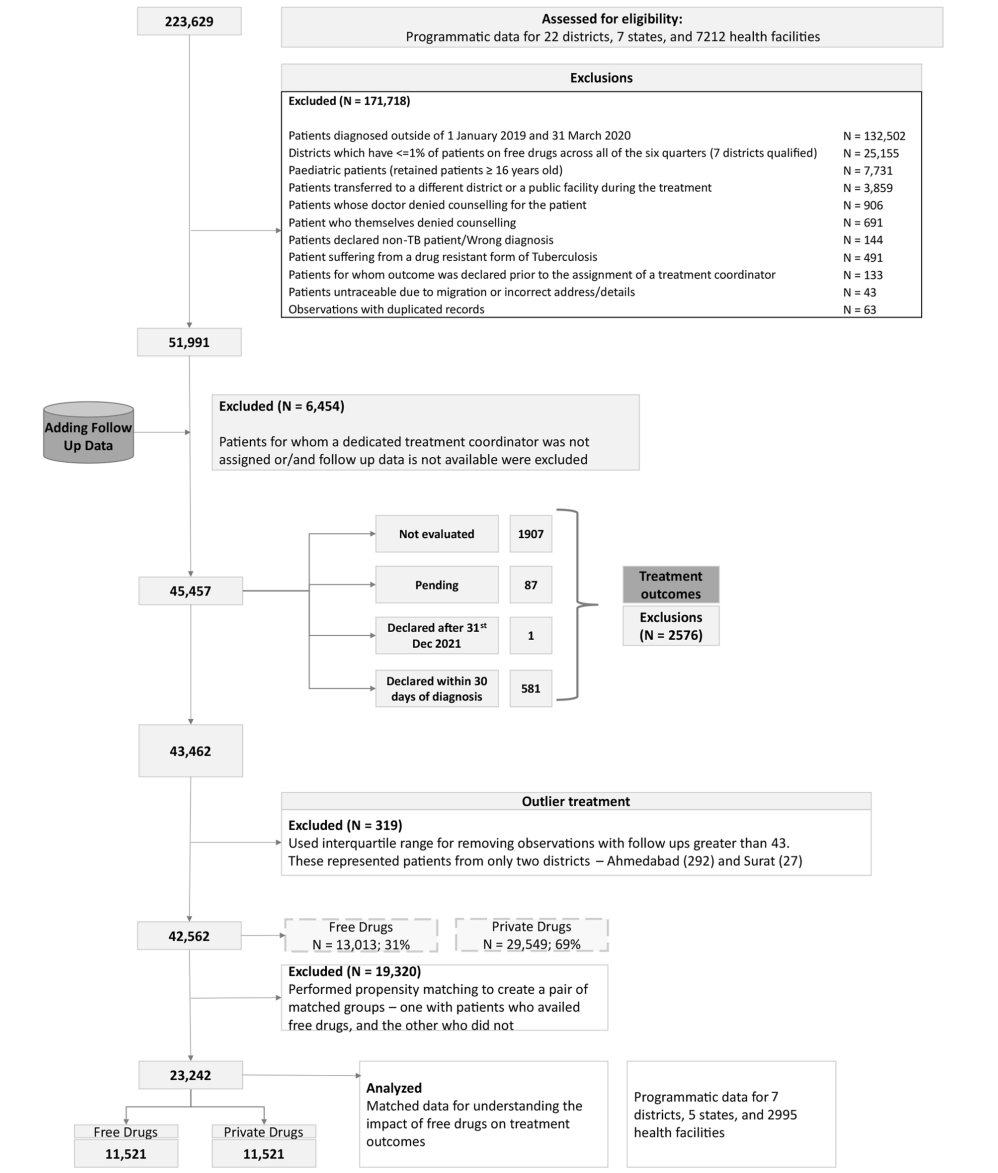
Data flow – selection criteria

Programmatic data recorded by JEET staff with records for more than 0.2 million patients diagnosed with TB across 22 cities in India, between 2019 and 2021 was used for the analysis (Table S1, Fig. 1). This data included patients diagnosed across 7,212 private facilities and includes (1) TB patients demographic characteristics such as age, sex, and diagnosing district, (2) TB diagnostic and treatment information, including type of diagnostic test performed, pulmonary or extrapulmonary diagnosis, whether free drugs were provided, patients’ treatment outcome, and number of follow-up contacts made by treatment coordinators.

### Inclusion & exclusion criteria

A total of 42,562 adult patients were deemed eligible for the analysis. A consort-style data flow diagram is provided to illustrate the data selection process (Fig. 1).

**Districts**: We considered data for patients from cities that began PPSA operations on or before 1 January 2019, and for whom at least 1% of patients availed of free drugs, for each of the 5 quarters of the study period. Seven of the 22 cities met these criteria: Ahmedabad, Bhopal, Darbhanga, Delhi, Gurgaon, Indore, and Surat.

#### Age & Study Period

Pulmonary and extrapulmonary drug sensitive adult TB patients (≥ 16 years) from these seven cities who began treatment between 1 January 2019 and 31 March 2020 and had a treatment outcome assigned on or before 31 December 2021 were considered. The study period ensures that enough time has passed for a treatment outcome to be recorded for both pulmonary and extrapulmonary patients. The study period also ensures that a large majority (99.63% or 42,406) of selected patients (42,562) were diagnosed with TB before the first lockdown was initiated in India (25th March 2020).

#### Availability of treatment coordinator information

Since our study is concerned with how the provision of free drugs impacted patient follow-up and treatment outcomes, we included only those patients who had a treatment coordinator assigned, along with data on number of follow-ups made by the treatment coordinator. The availability of data on follow-ups with a treatment coordinator indicates that the patient was actively under care for TB.

#### Availability of treatment outcome

Since our study is concerned with how the provision of free drugs impacted patient follow-up and treatment outcomes, we included only those patients who had a recorded treatment outcome. In some cases, however, a recorded treatment outcome does not automatically indicate if the patient completed their treatment (successfully or otherwise) or not. These include outcomes such as “not evaluated” and “lost to follow up”. Between these, we did not include patients whose outcomes were deemed as “not evaluated” – as that refers to cases transferred out to another treatment unit as well as cases for.

whom the treatment outcome is unknown to the reporting unit [21, 22]. We, however, include patients deemed as “lost to follow up”, as that refers to patients whose treatment was interrupted for more than a month [21, 22]. Other outcomes deemed ineligible include patients (a) who denied counselling, or whose doctors denied counselling for them, (b) who did not have an outcome declared until the end of the study, (c) who were transferred, (d) who were untraceable, and those (e) who were wrongly diagnosed.

#### Age criterion

We excluded paediatric patients (≤ 15 years of age) to account for the differences in care management and treatment regimens for paediatric patients compared to adult patients.

#### Drug resistant TB

Patients who were found to have a drug resistant form of TB were excluded from the analysis to account for the differences in care management and typically longer and more complicated treatment regimens.

#### Measurement Errors

We applied the following minimum criteria to our dataset to manage potential recording and measurement errors: (1) at least 30 days elapsed between a patient initiating treatment and being assigned a treatment outcome; (2) patients were assigned a treatment outcome after the assignment of a treatment coordinator.

#### Outlier treatment

**Fig. 2:**
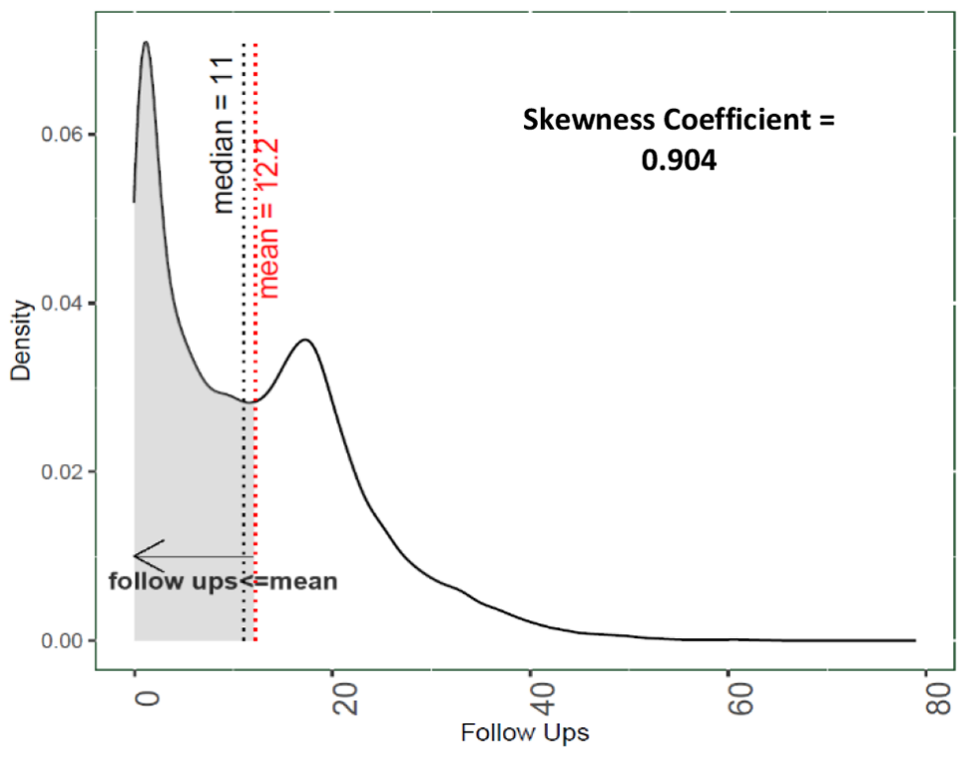
Density plot for successful follow ups made by the treatment coordinators N = 42,881

**Fig. 3:**
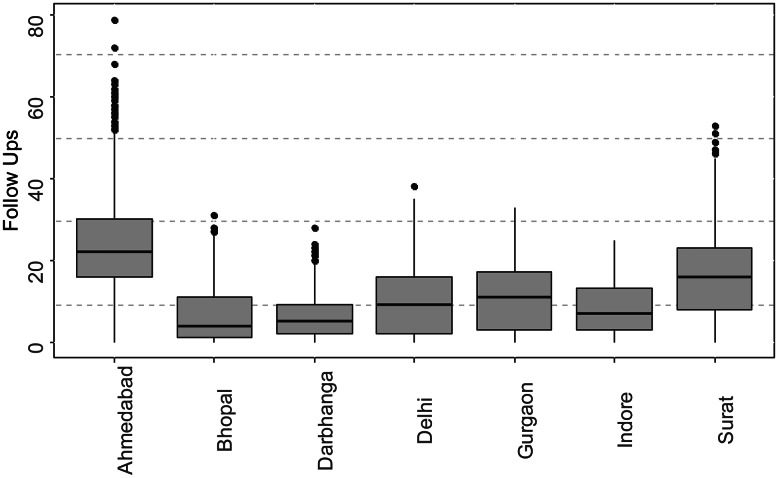
Box Plot for follow ups made by district, with marked outliers; N = 42,881

Programmatically, treatment coordinators were advised to make a total of 16 follow ups with the patient. Of these, 8 follow ups are recommended to be conducted in the initial 2 months or the intensive phase of the treatment (weekly), and 8 in the next four months or the continuous phase (fortnightly) of the treatment. An outlier treatment for the number of follow ups made with the patient was conducted to ensure our results do not get biased because of cases wherein a high number of follow ups can be attributed to a data error, or a clinical reason specific to a patients’s unique situation, or in some cases, a data entry error. The skewness coefficient for the data before the treatment (42,881 observations), was found to be 0.904 (Figs. 2 and 3), which reduced to 0.693 post the treatment. Outliers were identified using the interquartile range (IQR) criterion, following Seo [23] and Steven, [24]. The rationale is described in Appendix 2. A total of 319 (0.7%) patients were identified as outliers, having ≥ 44 recorded follow-ups. All of the outliers identified were from patients diagnosed in Ahmedabad (292 or 4% of the district’s patients) and Surat (27 or 0.4% of district’s patients). Conversations with TB program officers in these districts indicated the high likelihood of these values to be errors. We identified no outliers towards the lower range of the distribution.

### Outcomes of interest

Two primary outcomes were examined: (1) patient follow-ups and (2) treatment outcomes. Patient management typically involves a combination of factors relating to how the patient was supported by the health system throughout treatment. We considered the number of follow-ups made by treatment coordinators as a proxy for patient management, where more follow-ups translated into more engaged patient management. For treatment outcome, one of five outcomes were considered: (1) treatment complete, (2) cured, (3) treatment failure, (4) death, or (5) lost to follow-up. In the current study, we defined successful outcomes as either treatment complete or cured. The rest were considered as unsuccessful. A definition of each of the outcomes is provided in Appendix 8.

#### Propensity choice modelling

Since the study uses programmatic data, access to free drugs is not randomized among population groups, making it difficult to assess the average treatment effect (ATE) of free drugs on the outcomes of interest. While randomized experiments are typically utilized to understand the causal effect of a treatment, running such experiments is often cost intensive and laden with ethical issues, especially in studies concerning welfare and healthcare treatment effects [25]. Furthermore, an RCT was not feasible for the current study since free drugs are available to all TB patients in India under NTEP and are also widely prescribed in both the public and private sectors.

Several prior studies have acknowledged the usage of matching methods to infer causal insights from observational data, specifically in the field of health care assessment [26, 27]. Creating a dataset with observations matched on choice attributes provides an opportunity to estimate the average effect of the treatment as if it were a randomized experiment [28]. We used propensity score modelling to create a matched dataset that comprised of treated patients (free drugs) and untreated patients (no free drugs), which also included data on potential confounders for each individual [26, 29–32]. The propensity score was estimated for each patient, and then used to create comparable groups of people with access to free drugs (treated) and those who paid out-of-pocket (untreated). The scores were found to be adequate predictors of whether or not a patient had access to free drugs or not (Appendix 3). A combination of the nearest neighbor matching algorithm, along with exact matching was used to identify pairs of treated and untreated observations [33–36], and a total of five covariates were used for matching. This combination was used to ensure minimum possible bias. First, we employed a calliper width of 0.05 for the age and district variables, meaning the matched pairs were a maximum of 0.05 standard deviations away from each other, which is more conservative than the standard calliper of 0.2 [37]. Second, we employed exact matching rather than nearest neighbor matching on three variables: (1) proportion of males, (2) proportion of extra pulmonary cases, and (3) proportion of patients diagnosed using Xpert testing. Exact matching is preferable to nearest neighbor in many cases, but matching each individual on several independent variables results in a lower number of matched pairs in the final dataset [36, 38]. In our case, employing exact matching for selected variables resulted in 11,621 matched pairs in our final dataset, with only 11% (1,392) of treated observations going unmatched.

#### Statistical modelling

Using the matched dataset, we fit fixed-effects ordinary least squares (OLS) regression and fixed-effects logistic regression models to estimate the impact of free drugs on the number of follow-ups made with the patient and the odds of a successful treatment outcome, respectively. As covariates in the OLS regression model, we fitted a series of models, sequentially including free drug provision, sex of the patient, age category (16 to 19, 20 to 45, 46 to 65, and ≥ 66 years), TB type (pulmonary or extra pulmonary), and whether Xpert diagnostics were used. The logistic regression model was fit to assess the odds of a patient receiving a successful outcome at the culmination of treatment and utilized the same set of covariates.

The diagnosing district and diagnosing quarter were included as fixed effects in the OLS and logistic regression models to control for program-related influences of patient care and adherence to treatment. Treatment coordinator fixed effects were included to control for the impact of individual healthcare workers’ frequency and way of counselling patients, thereby impacting overall patient care and treatment outcomes. Only one of the two fixed effects – diagnosing district or treatment coordinator, could be included in a single model specification because of perfect correlation between them. Since a treatment coordinator provides care to patients diagnosed in their assigned district only, controlling for them enables controlling for district level fixed effects. The final model specification included double adjustment (relative to the propensity matching), based on the marginal improvement observed in the model fit.

To establish the linkage between follow ups and treatment outcomes, a logistic model was fit with follow ups as an additional dependent variable, while including for the status of free provision of drugs and other covariates.

#### Sensitivity analysis

Multiple sensitivity analyses were conducted to understand the impact of free drugs on follow ups and treatment outcomes. These are illustrated in the forest plots (Figs. 6 and 7), tabular results provided in Appendix 7. Our results were robust to specifications that excluded cases where treatment outcome resulted in lost to follow up, or when we ran district-specific models. They were also robust to including patient observations with treatment outcomes as (a) pending or (b) not evaluated. Here, we assumed the treatment outcomes to be unsuccessful. Since a significant share of our patients (55%) were undergoing treatment during the nationwide lockdown (outcomes declared after 25th March 2020); we ran sensitivity analyses for this patient group, as well as those who completed their treatment before the said date. The estimated coefficients were found to be significant. Finally, our results were also robust to alternative matching methods, including matching on the treatment coordinator, instead of matching on districts. This alternative matching specification resulted in 9,718 pairs, wherein 139 treatment coordinators had an equal share of patients being prescribed free drugs, and those that were paying out of pocket for drugs.

#### Statistical software

The analyses were conducted in R 2022.07.01. `MatchIt` package was used for the propensity score matching procedure, `broom`, `cobalt`, and `gtsummary` packages were used for visualizing fitted and residual values, generating balance plots from propensity choice modelling, and generating summary statistics, respectively. Packages used for data cleaning, preparing the analytical datasets, measuring skewness, and visualizing results were `dplyr`, `tidyr`, `moments`, and `ggplot2`. The `sandwich` package was used to compute heteroscedasticity-consistent robust standard errors.

## Results

**Fig. 4:**
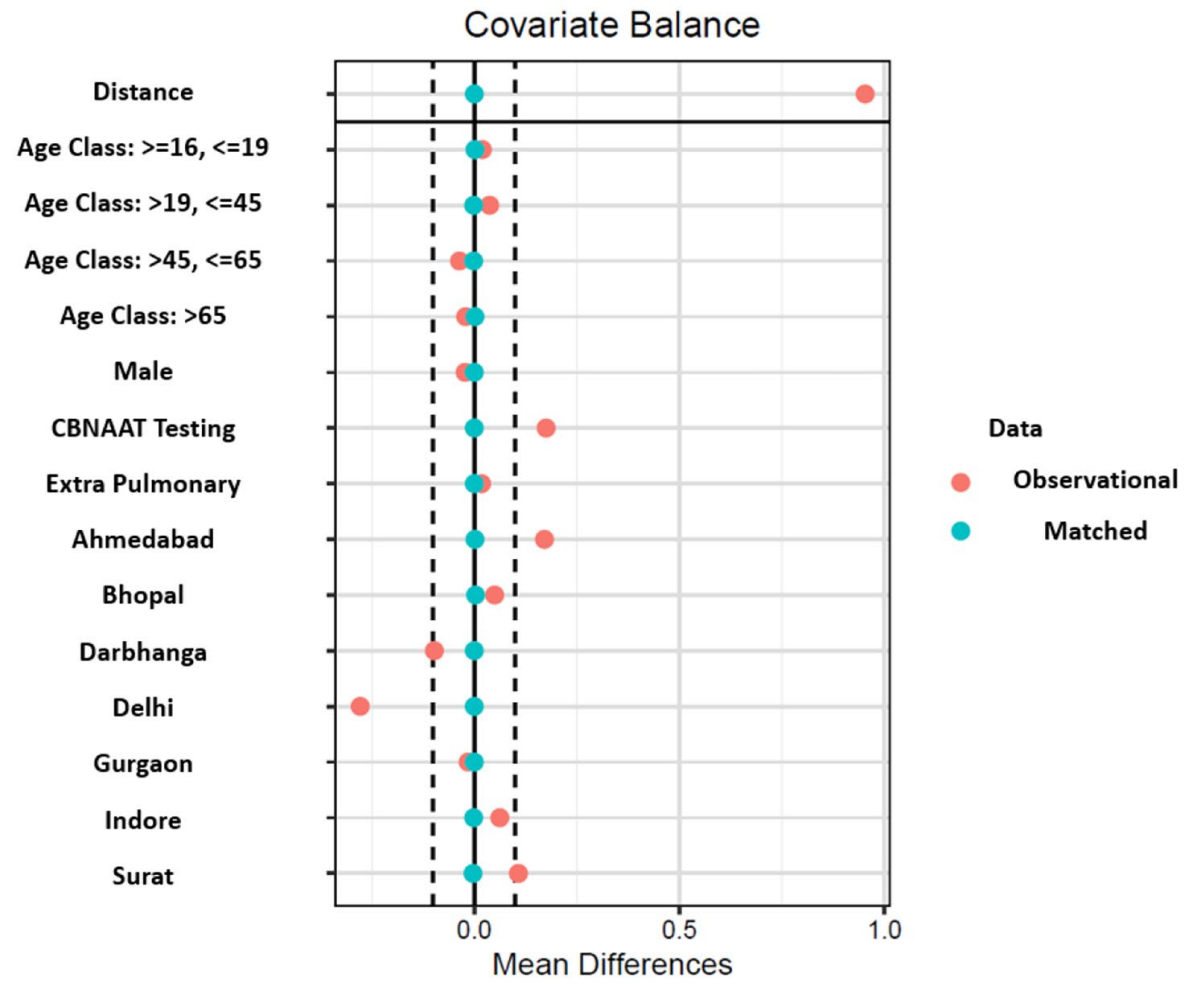
Results from balancing the covariates after the matching procedure Note: The red dots indicate the differences between standardized means of covariates in the matched and treated groups for the analytical or the unmatched dataset. The green dots indicate the same for the matched or the adjusted dataset.

**Fig. 5:**
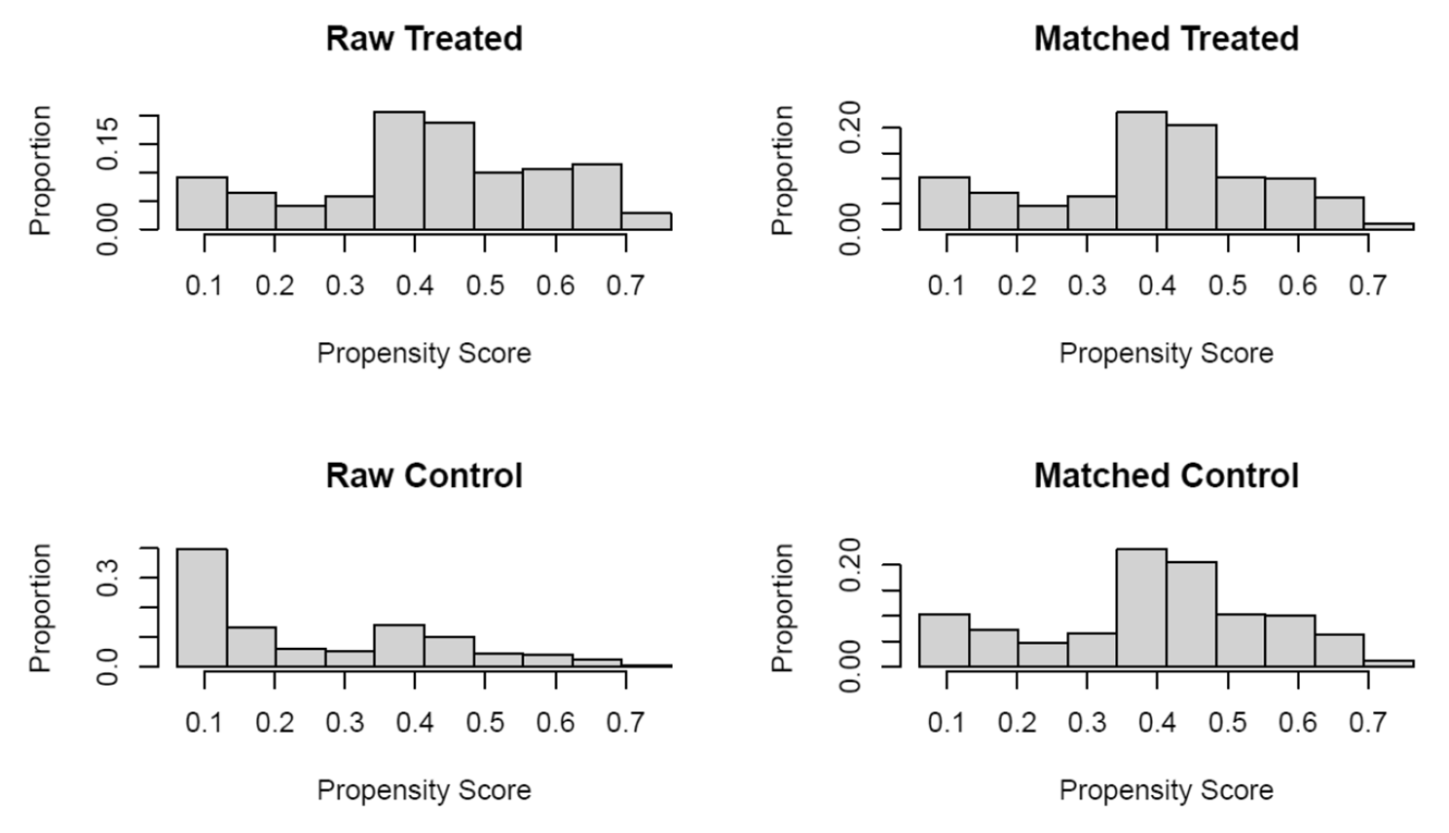
Propensity Scores, before and after the matching, in the treated (free drugs) and control groups (out of pocket drugs)

Tables 1, 2, 3 and 4 show the demographic and clinical profiles of patients in the dataset before matching (42,562 patients) relative to the matched dataset (23,242 patients or 11,621 pairs). The matching process using propensity scores brought the standardized propensity score difference between the treated and control group from 0.17 to 0, while balancing the mean difference between other covariates (Figs. 4 and 5; Appendix 4). Typically, we find patients to receive 11 follow ups across the duration of their treatment (Table 1, Observational Dataset). Patients in both the observational and matched dataset are found to have more follow ups if they are also receiving free drugs (Table 2). Patients with lost to follow up as a treatment outcome are observed to receive the least number (Median = 1) of follow ups (Table 3) and have the lowest share (14%) of patients on free drugs (Table 4). Patients with successful treatment outcomes (cured and treatment complete) have the highest share of patients receiving free drugs (Table 4). Treatment outcomes for patients receiving free drugs are found to be better across the five considered outcomes, relative to those who are buying out of pocket (Table 5). Among patients in the matched dataset, the mean and median age for the patient is 36 and 31 respectively, wherein 12,958 (56%) were male, and 6,074 (26%) of patients were diagnosed using Xpert testing (Table 1). There were 7,700 (33%) cases of extra pulmonary TB (Table 1, Matched Dataset). Overall, 21,883 (94%) of patients had a successful treatment outcome recorded (Table 1, Matched Dataset).

**Table 1:** Summary Statistics; before and after matching

	Observational dataset (N = 42,562)	Matched Dataset (N = 23,242)
**Males**	24,269 (57%)	12,958 (56%)
**Age Category**		
1. 16–19	4,621 (11%)	2,797 (12%)
2. 20–45	25,460 (60%)	14,470 (62%)
3. 46–65	9,759 (23%)	4,801 (21%)
4. >=65	2,722 (6.4%)	1,174 (5.1%)
**Age**		
Median (IQR)	33 (24, 50)	31 (23, 46)
Mean	37	36
**Follow Ups**		
Median (IQR)	11 (3,18)	14 (5,21)
Mean	11.91	14.27
**Access to free drugs**	13,013 (31%)	11,621 (50%)
**Xpert Testing**	8,833 (21%)	6,074 (26%)
**Extra Pulmonary**	13,212 (31%)	7,700 (33%)
**District**		
Ahmedabad	7,595 (18%)	5,924 (25%)
Bhopal	3,931 (9.2%)	2,995 (13%)
Darbhanga	4,547 (11%)	1,010 (4.3%)
Delhi	14,112 (33%)	3,614 (16%)
Gurgaon	2,262 (5.3%)	1,028 (4.4%)
Indore	2,595 (6.1%)	2,019 (8.7%)
Surat	7,520 (18%)	6,652 (29%)
**Diagnosing Quarter**		
2019 Q1	6,597 (15%)	3,389 (15%)
2019 Q2	9,538 (22%)	4,545 (20%)
2019 Q3	8,871 (21%)	5,025 (22%)
2019 Q4	8,314 (20%)	4,824 (21%)
**Successful Treatment Outcome**	39,439 (93%)	21,883 (94%)

***Note***: *(a) The table showcases the numbers segregated by free drug provision status, and within group percentages for them; (b) the p value for testing difference of means in groups with and without free drug provision; (c) n (%); Median (IQR) is given for continuous variables (age & follow ups); (d) Successful treatment outcome refers to cases where a patient has been assigned an outcome of “cure” or “treatment complete”; against the three other outcomes examined. Note that the share of successful outcomes is relative to the five outcomes examined and may not reflect the actual success percentage of treatment outcomes as assessed programmatically under the PPSA programs. Typically, the latter will be lower as patients with outcomes “not evaluated” or those who “denied counselling”, “are untraceable” are categorized as unsuccessful outcome, and these are not considered in our analysis*

**Table 2:** Summary Statistics; before and after matching by free drug provision status

	Observational dataset (N = 42,562)			Matched Dataset (N = 23,242)		
	*Segregated by free drug provision status*			*Segregated by free drug provision status*		
**Access to free drugs**	0, N = 29,549	1, N = 13,013	p-value	0, N = 11,621	1, N = 11,621	p-value
**Males**	17,046 (58%)	7,223 (56%)	< 0.001	6,479 (56%)	6,479 (56%)	> 0.9
**Age Category**			< 0.001			> 0.9
1. 16–19	3,030 (10%)	1,591 (12%)		1,390 (12%)	1,407 (12%)	
2. 20–45	17,335 (59%)	8,125 (62%)		7,241 (62%)	7,229 (62%)	
3. 46–65	7,104 (24%)	2,655 (20%)		2,409 (21%)	2,392 (21%)	
4. >=65	2,080 (7.0%)	642 (4.9%)		581 (5.0%)	593 (5.1%)	
**Age**			< 0.001			0.007
Median (IQR)	34 (24, 50)	30 (23, 46)		32 (23, 47)	30 (23, 46)	
Mean	38	36		36	36	
**Follow Ups**						< 0.001
Median (IQR)	8 (2, 16)	17 (8, 23)	< 0.001	11 (3, 19)	16 (8, 23)	
Mean	10	17		12	16	
**Xpert Testing**	4,512 (15%)	4,321 (33%)	< 0.001	3,037 (26%)	3,037 (26%)	> 0.9
**Extra Pulmonary**	9,013 (31%)	4,199 (32%)	< 0.001	3,850 (33%)	3,850 (33%)	> 0.9
**District**			< 0.001			0.3
Ahmedabad	3,724 (13%)	3,871 (30%)		2,952 (25%)	2,972 (26%)	
Bhopal	2,282 (7.7%)	1,649 (13%)		1,438 (12%)	1,557 (13%)	
Darbhanga	4,042 (14%)	505 (3.9%)		505 (4.3%)	505 (4.3%)	
Delhi	12,305 (42%)	1,807 (14%)		1,807 (16%)	1,807 (16%)	
Gurgaon	1,707 (5.8%)	555 (4.3%)		514 (4.4%)	514 (4.4%)	
Indore	1,238 (4.2%)	1,357 (10%)		1,022 (8.8%)	997 (8.6%)	
Surat	4,251 (14%)	3,269 (25%)		3,383 (29%)	3,269 (28%)	
**Diagnosing Quarter**			< 0.001			< 0.001
2019 Q1	5,068 (17%)	1,529 (12%)		2,042 (18%)	1,347 (12%)	
2019 Q2	7,133 (24%)	2,405 (18%)		2,392 (21%)	2,153 (19%)	
2019 Q3	6,153 (21%)	2,718 (21%)		2,582 (22%)	2,443 (21%)	
2019 Q4	5,462 (18%)	2,852 (22%)		2,283 (20%)	2,541 (22%)	
**Successful Treatment Outcome**	27,041 (92%)	12,398 (95%)	< 0.001	10,817 (93%)	11,621 (95%)	< 0.001

*Note: (a) The table showcases the numbers segregated by free drug provision status, and within group percentages for them; (b) the p value for testing difference of means in groups with and without free drug provision; (c) n (%); Median (IQR) is given for continuous variables (age and follow ups); Pearson’s Chi-squared test; Wilcoxon rank sum test are used for testing difference in groups; (d) Successful treatment outcome refers to cases where a patient has been assigned an outcome of “cure” or “treatment complete”. Note that the share of successful outcomes is relative to the five outcomes examined and may not reflect the actual success percentage of treatment outcomes as assessed programmatically under the PPSA programs. Typically, the latter will be lower as patients with outcomes “not evaluated” or those who “denied counselling”, “are untraceable” are categorized as unsuccessful outcome, and these are not considered in our analysis;*

**Table 3:** Mean and median number of follow-ups by treatment outcome for analytical dataset, before matching; N = 42,562

Treatment outcome	N	Mean follow-ups	Median follow-ups
Cured	152	6.6	5
Treatment complete	39,287	12.5	12
Died	1,415	6.7	5
Lost to follow-up	1,597	2.9	1
Treatment failure	111	5.4	2

**Table 4:** Free drug provision by outcome status for the analytical dataset, before matching; N = 42,562

Treatment outcome	N	% Patients on Free drugs
Cured	152	49%
Treatment complete	39,287	31%
Treatment failure	111	21%
Died	1,415	26%
Lost to follow-up	1597	14%

**Table 5:** Outcome rates by free drug provision for the analytical dataset, before matching; N = 42,562

Treatment outcome	Private drugs	Free drugs	% Point Change in outcome rates
Cured	0.3%	0.6%	0.3%
Died	3.5%	2.8%	-0.7%
Lost to follow-up	4.6%	1.7%	-2.9%
Treatment complete	91.2%	94.7%	3.5%
Treatment Failure	0.3%	0.2%	-0.1%

### Follow-ups with patients

**Fig. 6:**
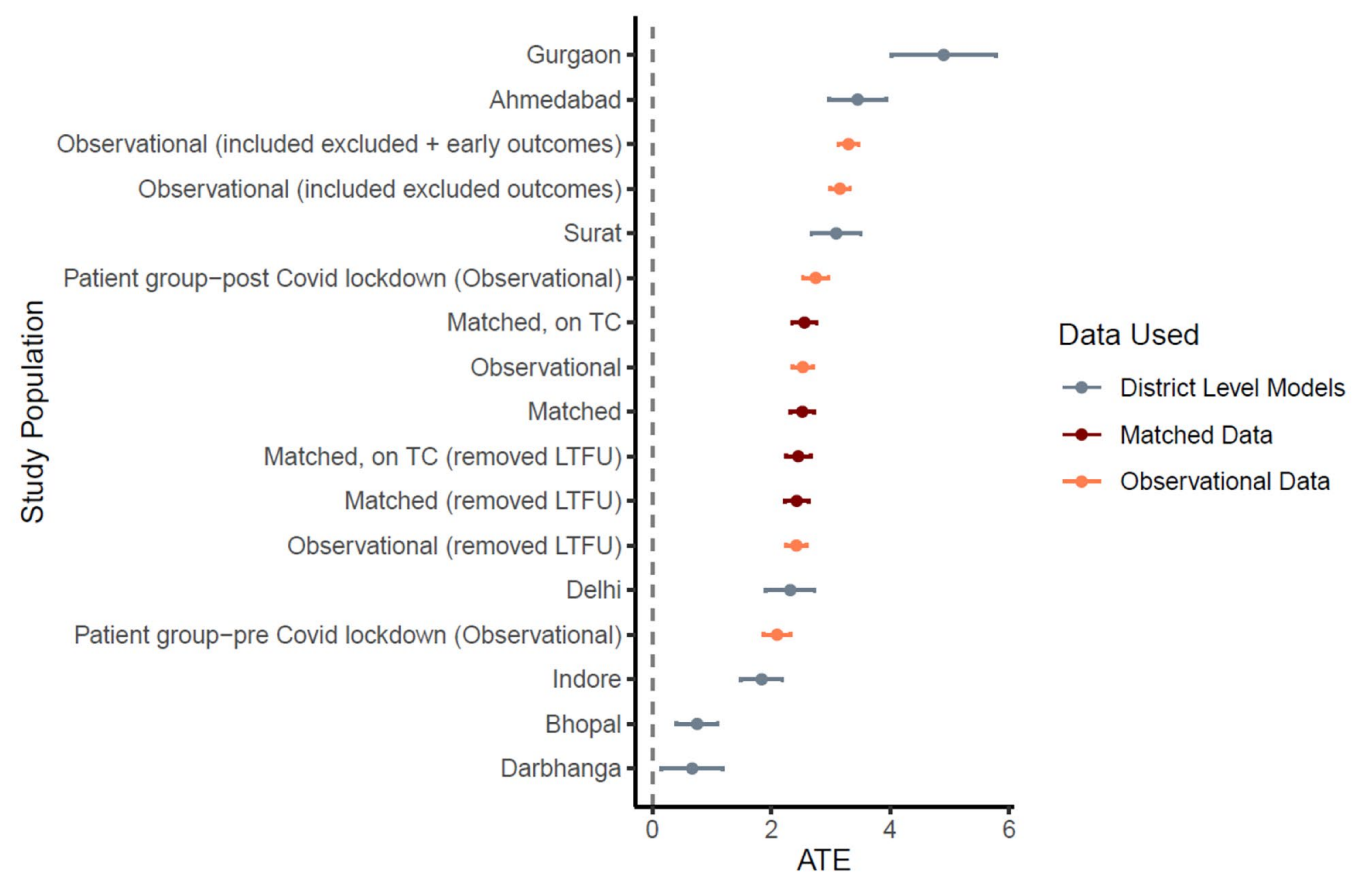
Forest Plot for OLS Regression; Impact of free drug provision on follow Ups Note: 1) LTFU refers to Lost to follow up; 2) Dotted line at X = 0 helps in visualizing the sub populations which reveal a significant impact (or not) of free drug provision on follow ups outcomes; 3) All district wise models are fitted on the matched dataset; 4) Matched on TC refers to a matched dataset which was created by matching on treatment coordinator, and not on district. This alternative matching specification ensures an equal share of patients receiving free drugs and not receiving free drugs and results in 19,436 observations or 9718 matching pairs; 5) Excluded outcomes refer to “not evaluated”, and “pending” outcomes, which were excluded in the base analysis – These were considered as unsuccessful for this sensitivity analyses; 6) Early outcomes refer to outcomes which were declared within 30 days of diagnosing date, and were excluded from the base analysis; 7) Patient groups, pre- and post-lockdown, refer to patients who had their treatment outcomes declared before and after 25th March 2020, respectively.

Within the matched dataset, patients on free drugs received more follow-ups from treatment coordinators (Mean = 16, Median (IQR): 16 (8, 23)) than patients who paid out of pocket for their drugs (Mean = 12, Median (IQR): 11 (3, 19)) (Table 2). We fit a series of six regression models that progressively added patient-level covariates, fixed-effects for diagnosing quarter, and diagnosing district. Model F fits best and includes fixed effects for treatment coordinator (Table 6). The model estimates an average treatment effect of 2.5 additional follow ups as a result of the free drugs (95% C.I. = 2.325 to 2.719). This is equivalent to a 25% (31%) increase in mean (median) follow-ups associated with receiving free drugs, when compared with patients who pay out of pocket (Mean = 10, Median = 8) (Table 2). These results were robust to the sensitivity analyses conducted by removing data for patients who were lost to follow-up as well as when we ran individual district-level models (Appendix 7 & Fig. 6).

**Table 6:** OLS regression model using matched dataset; dependent variable = number of follow ups made with the patient; N = 23,242

	Model A	Model B	Model C	Model D	Model E	Model F
Free drugs	3.933***	3.933***	3.333***	3.321***	2.504 ***	2.522***
95% C.I.	(3.675, 4.192)	(3.675, 4.191)	(3.082, 3.585)	(3.115, 3.527)	(2.306, 2.701)	(2.325, 2.719)
All Covariates		Yes	Yes	Yes		Yes
Diagnosing Quarter FE			Yes	Yes	Yes	Yes
Treatment Coordinator FE					Yes	Yes
District FE				Yes		
Observations	23,242	23,242	23,242	23,242	23,242	23,242
R2	0.037	0.042	0.114	0.409	0.509	0.512
Adjusted R2	0.037	0.041	0.114	0.408	0.505	0.509

**Note**: (a) 95% C.I. based on robust standard errors; (b) All models were fit on the matched dataset; (c) *p < 0.1; **p < 0.05; ***p < 0.01;

### Treatment outcomes

**Fig. 7:**
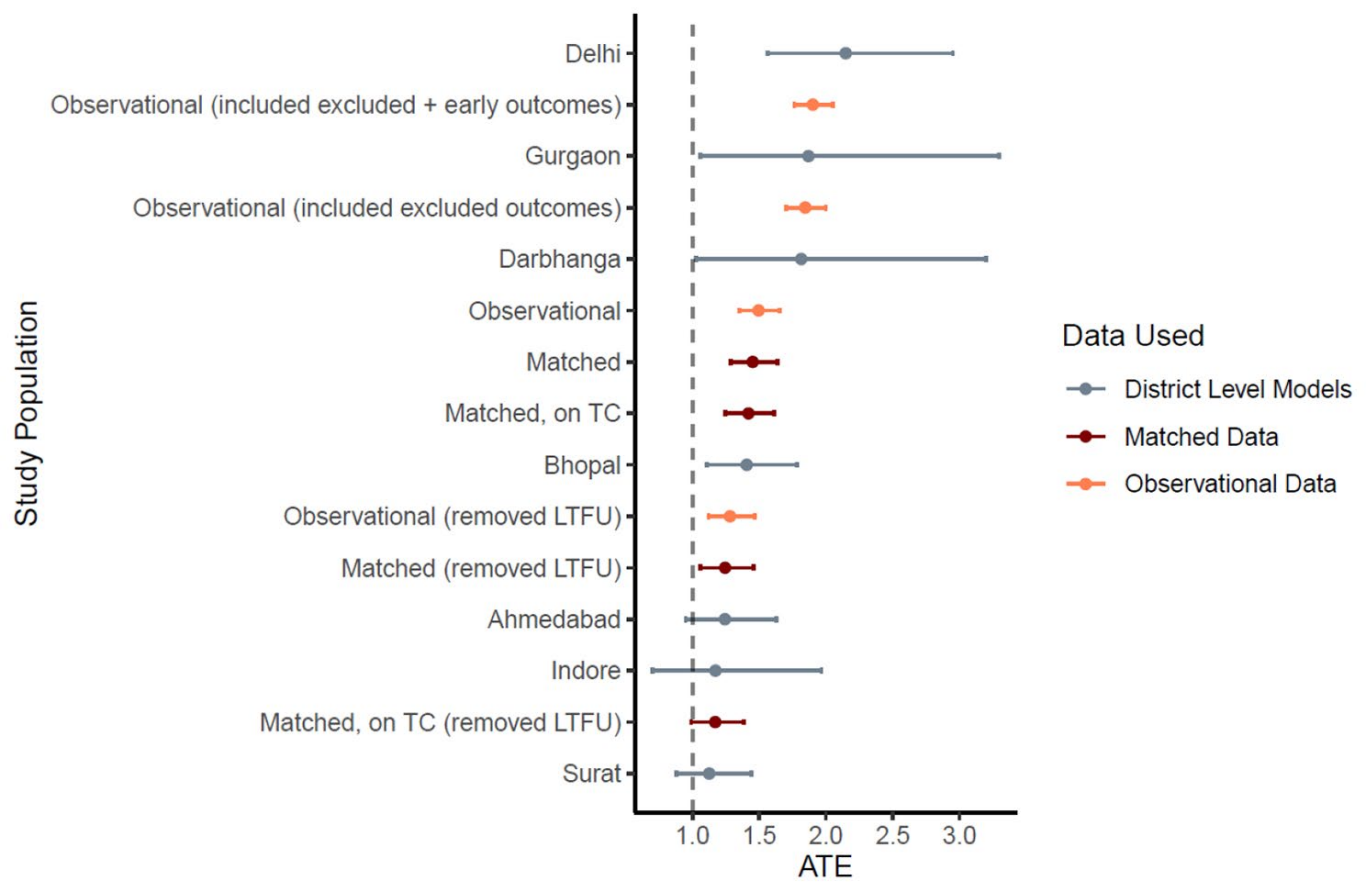
Forest plot; sensitivity analysis Note: 1) OR Ratios are displayed along with 95% C.I.; 2) Dotted line at X = 1 illustrates the sub-populations which reveal a significant impact of free drug provision on treatment outcomes (to the right) or not (to the left) 3) LTFU refers to lost to follow-up; 4) All district wise models are fitted on the matched dataset; 5) Matched on TC refers to a matched dataset which was created by matching on treatment coordinator, and not on district. This alternative matching specification ensures an equal share of patients receiving free drugs and not receiving free drugs and results in 19,436 observations or 9718 matching pairs; 5) Excluded outcomes refer to “not evaluated”, and “pending” outcomes, which were excluded in the base analysis – These were considered as unsuccessful for this sensitivity analyses; 6) Early outcomes refer to outcomes which were declared within 30 days of diagnosing date, and were excluded from the base analysis 7) Patient groups, pre- and post-lockdown, refer to patients who had their treatment outcomes declared before and after 25th March 2020, respectively.

Within the matched dataset, 95% (11,066) of those with access to free drugs had a successful treatment outcome, while 93% (10,817) who paid out-of-pocket had a successful outcome. A series of fixed-effects logistic regression models (Table 7) each revealed a statistically significant greater odds of a successful treatment outcome for patients who received free drugs compared to those who paid out of pocket. Model E fits best and reveals 45% higher odds (OR = 1.4519; 95% C.I. [1.288 to 1.637]) of a successful outcome for patients who received free drugs relative to those who paid out of pocket. The result is robust to multiple sensitivity analyses (Appendix 7 & Fig. 7).

**Table 7:** Logistic regression results showing impact of free drugs on treatment outcomes using matched dataset; N = 23,242

	Model A	Model B	Model C	Model D	Model E	Model F
Free drugs (Odds ratio)	1.482***	1.4945***	1.5028***	1.5364***	1.4381***	1.4519***
95% C.I.	(1.326, 1.657)	(1.336 1.672)	(1.342, 1.682)	(1.370, 1.722)	(1.278, 1.618)	(1.288, 1.637)
All Covariates		Yes	Yes	Yes		Yes
Diagnosing Quarter FE			Yes	Yes	Yes	Yes
District FE				Yes		
Treatment Coordinator FE					Yes	Yes
Observations	23,242	23,242	23,242	23,242	23,242	23,242
Log Likelihood	-5,152.60	-4,978.19	-4,976.30	-4,855.65	-4,861.64	-4,690.78
Akaike Inf. Crit.	10,309.20	9,972.39	9,976.60	9,747.30	10,021.30	9,691.57

**Note**: (a) 95% C.I. based on robust standard errors; (b) All models were fit on the matched dataset; b) *p < 0.1; **p < 0.05; ***p < 0.01

### Link between free drugs, follow ups, and treatment outcomes

Including follow ups as a covariate in the logistic model reduces the size and significance of the coefficient on free drugs (Table 8). It also reveals a statistically significant coefficient on the follow ups, estimating 17% increased odds of a successful outcome, for every unit increase in follow ups with the patient. Results from this specification (Table 8), along with the model revealing a significant impact of free drugs on follow ups (Table 6), lead us to conclude that free drugs are leading to better treatment outcomes, *primarily* through their impact on the number of follow ups made with the patient.

**Table 8:** Logistic regression results showing impact of free drugs on treatment outcomes using matched dataset; N = 23,242; including follow ups as a covariate

	Model A (Odds Ratio)	95% C.I.
Free drugs	0.977	(0.86, 1.110)
Follow ups (Odds Ratio)	1.171*	(1.158, 1.183)
All Covariates	Yes	
Diagnosing Quarter FE	Yes	
District FE	No	
Treatment Coordinator FE	Yes	
Observations	23,242	
Log Likelihood	-4101.450	
Akaike Inf. Crit.	8514.9	

**Note**: (a) 95% C.I. based on robust standard errors; (b) The model was fitted on the matched dataset; (c) *p < 0.1; **p < 0.05; ***p < 0.01

## Discussion

**Fig. 8:**
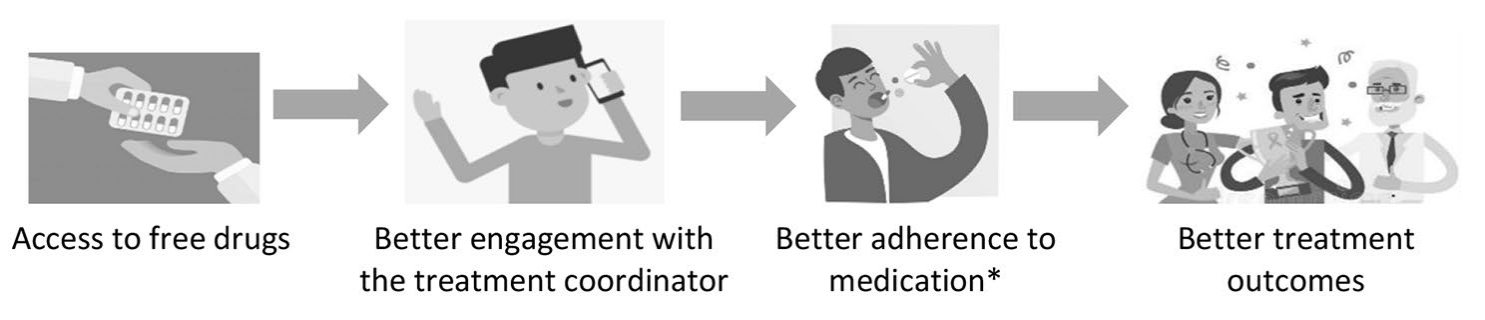
Theory of Impact of free drugs on patient management and treatment outcomes ***Note***: *This figure illustrates, in a simplified manner, the theory of impact – between the provision of free drugs, and their impact on patient management, and subsequently, their treatment outcomes. It does not include many factors, which may or may not be controlled for in our analysis, that affect program outcomes.* * Our study does not measure the actual impact on treatment adherence due to paucity of data available

To our knowledge, this is the first study that examines the impact of free drugs on TB patients seeking care in the private sector in India using a quasi-experimental approach. The analyses illustrate that free drug provision can act as an important policy tool in improving the odds of a successful treatment outcome, likely through increasing patient engagement with their treatment coordinators. This is illustratively shown in Fig. 8. The findings stay robust when tested on a balanced dataset, which was obtained after employing a strict propensity score matching method and controlling for different potential confounders. Our results stay robust across a heterogeneous group of 7 districts, which differ with respect to prevalence rates, healthcare systems, and regulatory environments, among other things.

### Impact of free drugs – on reducing cost, and increasing follow ups

The economic burden of TB treatment goes beyond the direct costs associated with drugs and physician’s consultation fee, and extends to the indirect costs associated with loss of income, leading many patients to face catastrophic expenditure [39], and therefore sell assets and seek loans to pay for their treatment [40, 41]. While our analysis does not measure if and how the availability of free drugs would have reduced the financial burden of the recipients, anecdotal evidence obtained to researchers from the ground, as well as earlier research, indicates that it would have alleviated at least part of the financial burden [42, 43].

Beyond the financial benefits, the study estimates a significant increase in the successful follow ups made between the patient and their designated treatment coordinator, for patients availing free drugs. Previous investigations of TB treatment engagement from China have demonstrated the importance of patient communication with healthcare providers, particularly surrounding patients’ desire for more information about TB and their treatment [44]. In the current study, we estimate that patients availing free drugs will receive 2.5 additional follow ups. This increase in the number of follow ups may have provided patients with the opportunity to not only ask questions about their treatment, but to also engage in the constellation of services offered by Project JEET and the NTEP. This aligns with earlier studies that have highlighted the role of treatment coordinators in the PPSA program, especially in light of poor counselling services generally offered by private sector provides and their varying degree of understanding of TB diagnosis and treatment guidelines [45]. The increased follow ups may have also helped patients in forming linkages to social services and other supports facilitated by Project JEET, further enabling patients’ ability to remain engaged in care [46].

### Impact of free drugs on treatment outcomes

We also estimate 45% higher odds of treatment success, for both the observational and matched datasets, varying but largely staying significant across a differentiated group of districts. Descriptively, we see that patients on free drugs have lower death rates (2.8% for patients on free drugs vs. 3.5% for patients on private drugs) and lower lost to follow up (1.7% vs. 4.7%). While the incremental impact of such an intervention might vary in alternate settings, the robustness of results across different population settings is strong evidence to use similar interventions for linking patients to other social support services by way of increased dialogue with the relevant healthcare professionals. Access to similar support mechanisms has been previously associated with increased treatment success. For instance, TB patients experienced a 10-percentage point decrease in treatment dropout when enrolled in a community-based social support program in Ukraine, a country with a growing degree of drug resistant TB, fuelled by high default rates [47]. In Brazil, studies using propensity score matched datasets showed that TB patients receiving a government provided monthly stipend experienced a 7–11% increase in TB success rates [48, 49].

### Impact of follow ups on patient engagement and treatment outcomes

While further research is warranted to understand the mechanisms in action during the follow-up visits, attention on supportive services for private-sector TB patients, where free drugs are commonly dispensed with minimal government oversight, is growing and acknowledged as a cornerstone to enabling India to meet its elimination target [18, 50]. Studies evaluating the role of digital support in India have further reinforced the need for psychosocial support for patients and their family members – from increased human interaction, notwithstanding the increasing role of digital interventions [51, 52]. In our study, for patients availing free drugs, the enhanced follow-up schedule could have allowed them to resolve their questions and concerns about the TB treatment, while also reducing any misconceptions and arresting the spread of misinformation. For instance, ADRs are common among TB patients and are a driving reason for defaulting on the treatment [53, 54]. Patient interactions with treatment coordinators may have encouraged adherence, in the face of these or/and other challenges. Additionally, consistent contact with a treatment coordinator, may also have helped patients manage the physical and mental burden of TB and potentially contributed to expediting access to any additional clinical support required.

### Impact of COVID lockdown on patient services

We note that 55% of our patient population from within the analytical dataset (42,562) was undergoing treatment when the nationwide COVID lockdown was announced in India (24th March 2020). It is likely that this would have impacted the services availed by patients, including free drugs and follow ups. This analyses, in depending largely upon retrospective programmatic data, cannot precisely assess how this impacted the patients’ treatment, or/and even diagnosis. However, sensitivity analyses illustrated an enhanced impact of free drugs on the population undergoing treatment during the lockdown. It is also worth noting that the share of patients availing free drugs significantly increased as the PPSA programs matured (Table S12). This is, at least, partly driven by the enhanced engagement of private providers by JEET staff – which would have increased the number of patients they prescribed free drugs. While further research is warranted to understand if and how patient services are impacted during a time of crisis, our results do indicate the ability of such an intervention to positively affect program outcomes in diverse systemic and regulatory environments.

## Conclusions

Our study employed a robust quasi-experimental approach to construct a highly comparable control group and demonstrated a meaningful impact of free drugs on patient follow-ups and treatment success among private sector TB patients. While previous studies have highlighted the role of continuous and effective patient management, challenges exist both with respect to the resources needed for programs to fully support patients as well as patients’ responsiveness to this support.

For the former, these results provide a strong argument to extend support services to larger populations of patients, justifying increased expenditure and capacity building for such initiatives. With respect to managing patients’ varying responsiveness to such support, a deeper behavioural design approach to implementing such support such initiatives is warranted. However, the study demonstrated that free drugs acted as a catalyst in increasing this engagement, possibly due to the logistical construct of treatment coordinators facilitating the refilling of prescription every 28 days. This suggests that similar interventions, such as provision of monthly benefit transfers for nutrition support, or a free health check-up, which requires the patient to speak with a healthcare professional to avail these benefits, can be instrumental in increasing patients’ awareness to their healthcare, provide an augmented opportunity to resolve their questions about the treatment and related challenges, improve adherence to treatment, thereby leading to better outcomes. These findings are easily translated to programs where a dedicated treatment coordinator might not be available, thus increasing the marginal benefit of such interactions. They are also applicable to other ailments where the treatment spreads over an extended period of time, or/and involves a complicated drug regimen, increasing the probability of challenges including but not limited to ADRs and forgetfulness in taking medication.

While our study setting is based on patients seeking care in PPSA engaged facilities in the private sector, our results stay robust across distinct geographies, spread across the northern, central, and western belts of India. The geographies not only differ with respect to TB prevalence rates, but also represent a diverse group of programmatic and regulatory conditions, which suggest the applicability of findings to a wider set of health-system settings and geographies.

Finally, the private healthcare sector is an important component of TB treatment in India, but it remains fragmented, and patients often go unsupported. Increasing the level of support private sector patients receive, such as those offered through Project JEET, facilitates treatment completion and aids patients in accessing the social services they need. However, we observe that a significant proportion of patients, even though diagnosed and treated under Project JEET facilities, were not provided with free drugs, a support available free of cost to all TB patients in India. Efforts to sensitize private providers about the benefits of providing free drugs, even in situations where the provider deems the patient as having the resources to buy these out of pocket, should be helpful in increasing patient care and treatment outcomes. Similar initiatives that pair effective clinical treatments with social support for private sector patients will prove invaluable as India progresses towards TB elimination.

### Limitations

There are several limitations to this analysis that warrant mention. **First**, our study is based on patients seeking care in PPSA engaged facilities within the private sector. This might have placed patients under a certain advantage relative to other patients seeking care in the private sector, in terms of receiving counselling or other services such as Xpert diagnostics or knowledge of additional services provided by NTEP (e.g., free drugs, direct benefit transfers) through contacts with their designated treatment coordinator. This limits the plausibility of extending the inferences drawn to other population settings. On the other hand, considering that this group might already be in an advantageous position and the intervention was still estimated to lead to better treatment outcomes for the group indicates that the benefits estimated are on the conservative side. Nevertheless, further research into the impact of interventions on patient linkages is warranted. **Second**, while our models present a strong fit with the covariates used, there are several additional and missing factors which would impact program outcomes, e.g., comorbidities, a patient’s socio-economic situation, or/and social support received by family and friends. In the absence of sufficient data, this study cannot ascertain how they would have impacted the final model coefficients. Additional research on how these factors impact a patient’s engagement with the system should provide more precise answers into scaling similar interventions in other population and regulatory settings. **Third**, our analyses rely on a binary indicator for patients’ availing free drugs or not. In reality, there could be patients who are availing free drugs, but then shift to private drugs, or vice-versa. There could be multiple reasons for this, including advice by the provider, or non-availability of free drugs at different time periods/geographies. The requisite data for the same was not maintained sufficiently accurately to add this layer of detail to our analysis. **Fourth**, it may also be noted that the final model specification used for drawing inferences is based on a matched dataset, and yet, controls for variables which were already balanced during the propensity matching process. This inculcates a double adjustment, which might not be considered necessary by some econometricians. However, we chose this model based on the practical motivations behind this analysis. Our objective is to emphasize the strong link between the availability of an intervention (such as free drugs) to positively impact patient engagement (follow ups with the treatment coordinator), and consequently treatment outcomes. Considering we do not have access to all possible variables affecting the number of follow ups made with the patient, choosing the model with the best fit is an attempt to highlight the directional impact of such an intervention. Additionally, double adjustment might not be ideal if our sample size were small as it could lead to lower precision of the treatment effect (the regression coefficient against free drugs). However, that is not the case with our observed sample. It is also worth noting that in our analysis, double adjustment led to no practical changes in the coefficients, or any resulting programmatic implications. The models without double adjustment (Model E in Tables 5 and 6), as well as some alternative specifications, are provided for the readers’ consideration. **Fifth**, the study uses a derived dichotomous outcome variable, wherein unsuccessful outcomes included treatment failure, death, and lost to follow-up, each of which may have their own risk profiles. Sensitivity analysis excluding patients with lost to follow-up as a treatment outcome (Appendix 7) supported the primary findings with high statistical significance. **Sixth**, all patients who had a treatment interruption greater than one month in duration are considered as being lost to follow-up. However, it cannot be determined if patients continued the treatment later, and if so, whether they were able to complete the treatment with a positive outcome. Hence, including lost to follow-up has the potential to bias these results. Some previous studies have not included lost to follow-up in their analyses for similar reasons [48]. However, to remain conservative, we adhered to the baseline criteria of including all patients who were under the active management of a treatment coordinator and had their outcomes reported at least a month after the date of diagnosis. Additionally, lost to follow-up makes up 3.8% (1,604) and 2.7% (614) of our analytical and matched datasets, respectively. Including lost to follow-up in analysis where these cases make less than < 5% of the overall population generally leads to little bias [55]. Regardless, further research is warranted to fully understand the differential risk profile of private sector TB patients, including the drivers of lost to follow-up and treatment failure. **Seventh**, we have excluded patient observations for which an outcome was either pending or not evaluated. If we compare to the overall observational sample used for analysis (42,562), “pending” and “not evaluated” cases make up 0.2% and 4.8% of our sample, respectively. Following the same inference as above (< 5% of our sample), excluding them should lead to little or no bias. Additionally, sensitivity analysis including these patients (Appendix 7) supported the primary findings with high statistical significance. **Eighth**, the majority of patients reported treatment completion, which was based on the provider declaring that patient need not take any more medications. Since cure rates are low due to lack of smear testing in the private sector, the metric of successful treatment completion itself has certain limitations. **Lastly**, while the results are largely consistent for different sub-populations analysed, a deeper understanding into how the impact of free drugs differs across varying socio-demographic profiles of patients, in different geographies and programmatic environments is required. For instance, the impact of free drugs on follow-ups differed significantly among districts, warranting further investigations into the contextual factors that influence the impact of free drugs.

## Electronic supplementary material

Below is the link to the electronic supplementary material.

Supplementary Material 1

## Data Availability

The datasets used and/or analysed during the current study are available from the corresponding author on reasonable request.

## **List of abbreviations**

ADR: Adverse drug reaction
ATE: Average treatment effect
CI: Confidence interval
FDC: Fixed dose combination
IQR: Interquartile range
JEET: Joint Effort for Elimination of Tuberculosis
OLS: Ordinary least squares
PPSA: Private Provider Support Agency
TB: Tuberculosis
NTEP: National Tuberculosis Elimination Program
NSP: National Strategic Plan for Elimination of Tuberculosis
