# Supplementary material for: Free drug provision for tuberculosis increases patient follow-ups and successful treatment outcomes in the Indian private sector: a quasi experimental study using propensity score matching
Source: BMC Infect Dis. 2023 Jun 21;23:421. doi: 10.1186/s12879-023-08396-5 (PMC10283201; doi:10.1186/s12879-023-08396-5)
Supplement: Supplementary file 1 — Supplementary Material 1 [file 12879_2023_8396_MOESM1_ESM.docx]

**Supplementary Text**

“Free drug provision for tuberculosis increases patient follow-ups and successful treatment outcomes in the Indian private sector: A quasi experimental study using propensity score matching”

Ridhima Sodhi^1^, Michael J. Penkunas,^2^ Arnab Pal^3^

1. Independent Consultant, Clinton Health Access Initiative, Inc., India
2. Independent Consultant, Clinton Health Access Initiative, Inc., Boston, MA, USA
3. Associate Director, Diagnostics, Surveillance and TB, Clinton Health Access Initiative, Inc., India

# Appendix 1 – Baseline Summary Statistics

Table S1 summarizes the number of patients diagnosed between Jan 2019 and December 2021 who were enrolled with Project JEET under the William J. Clinton Foundation in the seven states (22 cities) running the PPSA program.

**Table S1**

*Patients diagnosed of Tuberculosis between Jan 2019 and December 2021 across 7 states and 22 PPSA cities managed by WJCF*

| **state** | **district** | **#patients** | **#treatment coordinators** | **minimum diagnosing date** | **max diagnosing date** | **% patients on free drugs** |
| --- | --- | --- | --- | --- | --- | --- |
| Bihar | Darbhanga | 15,276 | 22 | 1/1/2019 | 12/30/2021 | 17% |
| Bihar | Madhubani | 2,775 | 5 | 4/13/2020 | 12/30/2021 | 33% |
| Bihar | Muzaffarpur | 5,709 | 6 | 9/1/2019 | 12/31/2021 | 26% |
| Bihar | Purba Champaran | 8,292 | 6 | 9/1/2019 | 12/30/2021 | 36% |
| Bihar | Purnia | 6,329 | 5 | 9/1/2019 | 12/30/2021 | 40% |
| Bihar | Saran | 5,098 | 5 | 9/2/2019 | 12/31/2021 | 44% |
| Delhi | Delhi | 41,728 | 76 | 1/1/2019 | 12/31/2021 | 18% |
| Gujarat | Ahmedabad | 25,186 | 46 | 1/1/2019 | 12/31/2021 | 49% |
| Gujarat | Surat | 22,090 | 25 | 1/1/2019 | 12/31/2021 | 49% |
| Haryana | Faridabad | 3,035 | 1 | 7/1/2020 | 12/27/2021 | 23% |
| Haryana | Gurgaon | 8,559 | 10 | 1/1/2019 | 12/31/2021 | 34% |
| Haryana | Rohtak | 2,819 | 5 | 1/1/2020 | 12/31/2021 | 22% |
| Madhya Pradesh | Bhopal | 9,741 | 13 | 1/1/2019 | 3/27/2021 | 45% |
| Madhya Pradesh | Indore | 8,492 | 15 | 1/1/2019 | 3/31/2021 | 45% |
| Rajasthan | Ajmer | 3,896 | 4 | 6/1/2020 | 12/30/2021 | 12% |
| Rajasthan | Bikaner | 4,360 | 5 | 10/1/2019 | 12/30/2021 | 20% |
| Rajasthan | Jaipur | 28,120 | 23 | 1/1/2019 | 12/31/2021 | 6% |
| Rajasthan | Jodhpur | 4,072 | 12 | 5/10/2020 | 12/31/2021 | 16% |
| Rajasthan | Kota | 4,387 | 9 | 5/15/2020 | 12/31/2021 | 13% |
| Rajasthan | Sikar | 5,564 | 4 | 10/1/2019 | 12/31/2021 | 22% |
| Rajasthan | Udaipur | 4,290 | 7 | 5/15/2020 | 12/25/2021 | 17% |
| Tamil Nadu | Chennai | 3,811 | 31 | 1/1/2019 | 3/29/2021 | 12% |
| Grand Total | | 223,629 | 335 |  |  |  |

Table S2 details the decisions taken at each stage to arrive at the final analytical dataset consisting of 42,562 patients, who were diagnosed across seven districts in India. This dataset was then used for matching using propensity scores.

**Table S2**

*Data selection process, detailed step wise*

| **#Patients** | **% Retained at each stage** | **Data Selection Criterion at each stage** |
| --- | --- | --- |
| 223,629 | 100.00% | Programmatic data for 22 districts, 7 states, and 7212 health facilities |
| 91,127 | 40.75% | Patients diagnosed between 1 January 2019 and 31 March 2020 |
| 65,972 | 72.40% | Districts which have patients (>=1%) on free drugs across all of the six quarters (7 districts qualified) |
| 58,241 | 88.28% | Patients ≥ 16 years old |
| 54,382 | 93.37% | Patients not transferred to a different district or a public facility during the treatment |
| 53,476 | 98.33% | Doctor has not denied counselling for the patient |
| 52,785 | 98.71% | Patient has not denied counselling |
| 52,661 | 99.77% | Patient is not declared a non-TB patient post diagnosis |
| 52,139 | 99.01% | Patient is not suffering from a drug resistant form of Tuberculosis (includes patients whose regimen is said to be changed) |
| 52,006 | 99.74% | Outcome was declared post the assignment of a treatment coordinator |
| 51,963 | 99.92% | Excluding patients who are untraceable due to migration or incorrect address/details |
| 51,900 | 99.88% | Excluding duplicated records |
| 45,457 | 87.59% | Follow-up data available for patients by a designated treatment coordinator |
| 43,550 | 95.80% | Excluding patients whose treatment outcomes is "not evaluated" |
| 43,463 | 99.80% | Excluding patients with "pending treatment outcome" |
| 43,462 | 100.00% | Outcome declared before 31 December 2021 |
| **42,881** | 98.66% | Outcome declared at least 30 days post the date of diagnosis |
| **42,562** | 93.63% | Post outlier treatment for follow ups. This retained patients who had received ≤ 43 follow ups |

# Appendix 2 - Outlier Treatment for Follow Ups

Pearson’s method was followed to find skewness. The `moments` package in R was used to compute skewness, and ggplot2 was used to visualize distributions.

$$Moment Coefficient of Skewness= \beta_{1}=\frac{m_{3}^{2}}{m_{2}^{3}}$$

where,

- $m_{2}=\frac{1}{n}\sum_{i=1}^{n} \left( x_{i}-\bar{x} \right)^{2}$*, second sample central moment*
- $m_{3}=\frac{1}{n}\sum_{i=1}^{n} \left( x_{i}-\bar{x} \right)^{3}$*, third sample central moment*

Here, $x_{i}$ represents an individual value of the variable in question and $\bar{x}$ defines the mean of that value.

Figures SF1 visually show the skewness for the overall data, and particularly for Ahmedabad and Surat in the state of Gujarat. A tabular distribution of number of follow-ups for each district can be seen in Table S3. Since the distribution is found to not follow a normal distribution (Figure SF2), we employed the interquartile range (IQR) criterion to identify the outliers. Since this method is based on the median, it is a better choice for identifying outliers in a skewed distribution such as this [1, 2]. Iglewicz and Hoaglin [3] detail a range of methods for outlier identification and testing in their textbook. Our choice of method is based on its relative simplicity and the efficacy of application for our particular data and context.

According to this criterion, all observations above q_0.75_ + 1.5*IQR or below q_0.25_ – 1.5*IQR are considered as potential outliers. Here, q_0.25_ and q_0.75_ refer to first and third quartile respectively, and IQR is the difference between the third and first quartile. As per this criterion, observations outside the following interval were considered as potential outliers:

$$I=[\text{q}_{\text{0.25 }}-1.5*IQR; \text{q}_{\text{0.75 }}+1.5*IQR]$$

A total of 319 (0.7% of 43,092) observations were found to be outliers using these methods, all of which pertained to patients in Ahmedabad & Surat.

**Figure SF1: Density plot overlaid on histogram for successful follow ups; by district**; N= 42,881


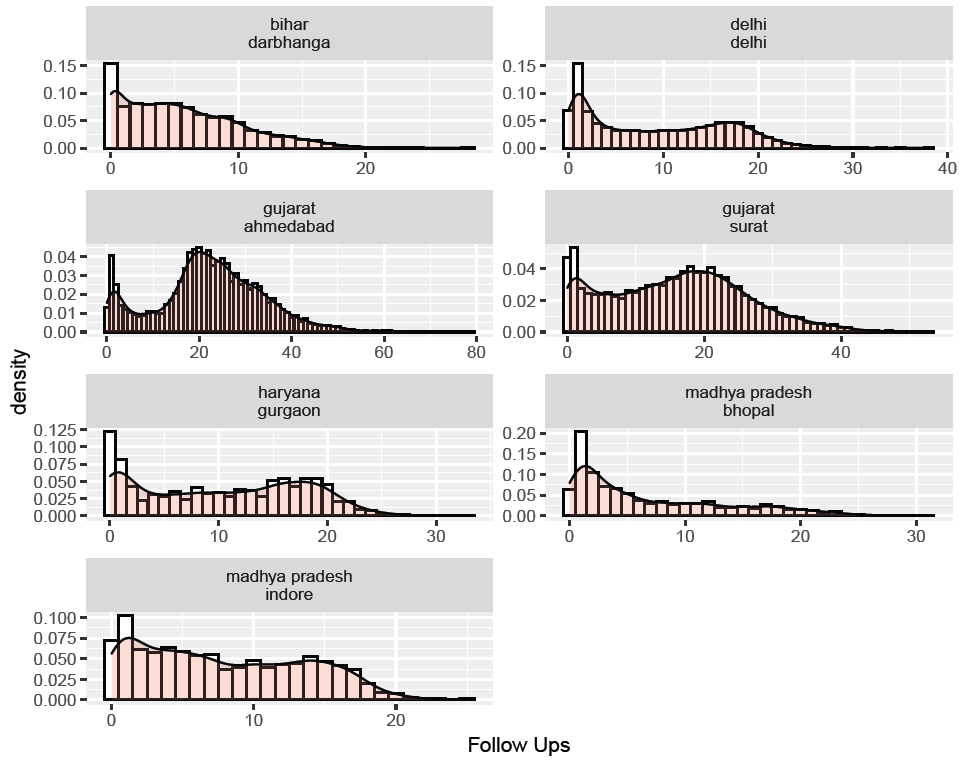

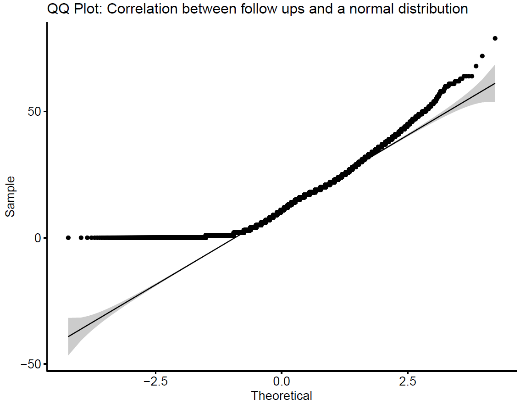


**Figure SF2: QQ Plot for follow ups**; N= 42,881

**Table S3**

*Share of patients receiving different number of follow ups, across different cities*

| **District** | **None** | **Between 1 to 12** | **Between 13-24** | **Between 25-36** | **Between 37-43** | **Between 44-48** | **≥49** | **District totals** |
| --- | --- | --- | --- | --- | --- | --- | --- | --- |
| **Ahmedabad** | 1% | 17% | 40% | 31% | 7% | 1.97% | 1.74% | 7,887 |
| **Bhopal** | 6% | 72% | 20% | 1% | 0% | 0.00% | 0.00% | 3,931 |
| **Darbhanga** | 15% | 75% | 9% | 0% | 0% | 0.00% | 0.00% | 4,547 |
| **Delhi** | 7% | 56% | 36% | 1% | 0% | 0.00% | 0.00% | 14,112 |
| **Gurgaon** | 12% | 44% | 42% | 1% | 0% | 0.00% | 0.00% | 2,262 |
| **Indore** | 7% | 66% | 26% | 0% | 0% | 0.00% | 0.00% | 2,595 |
| **Surat** | 5% | 33% | 42% | 17% | 2% | 0.30% | 0.06% | 7,547 |
| *Note: The column titles indicate the number of follow-ups, while the value in rows indicates the share of patients who received the said number of follow-ups. District totals are given in the last column* | | | | | | | | |

# Appendix 3 –Estimating propensity scores

We utilized propensity score modelling [4–6] to create a matched dataset comprised of treated patients (free drugs) and untreated patients (no free drugs), by means of a logistic regression model, including all available potential confounders. The exact model results from the same are given in Table S4, and Table S5 displays the mean propensity scores between patients who actually received free drugs or not. The accuracy of the model is 70.6% with a specificity rate of 23.2%. Figure SF3 shows the distribution of propensity scores visually, segregated by whether or not the patient received free drugs

**Table S4**

*Estimating propensity score using logistic regression; N = 42,562*

| **Dependent Variable: free drugs** | |
| --- | --- |
| Male | 0.919*** (0.872, 0.965) |
| Age: 20-45 | 0.804*** (0.731, 0.877) |
| Age: 46-65 | 0.655*** (0.572, 0.739) |
| Age: > =65 | 0.563*** (0.445, 0.681) |
| Xpert testing | 2.454*** (2.400, 2.507) |
| Extrapulmonary | 1.198*** (1.147, 1.248) |
| Bhopal | 0.658*** (0.578, 0.737) |
| Darbhanga | 0.114** (0.009, 0.219) |
| Delhi | 0.147*** (0.079, 0.215) |
| Gurgaon | 0.352*** (0.245, 0.460) |
| Indore | 0.924*** (0.832, 1.016) |
| Surat | 0.682*** (0.616, 0.748) |
| Constant | 1.105*** (1.020, 1.190) |
| Observations | 42,562 |
| Log Likelihood | -22,501.40 |
| Akaike Inf. Crit. | 45,028.80 |
| Note: a) 95% C.I. based on robust standard errors; b) *p<0.1; **p<0.05; ***p<0.01 | |
|  |  |

**Table S5**

*Propensity Score estimated by whether or not a patient received free drugs*

| **status** | **N** | **Mean score** | **Median score** |
| --- | --- | --- | --- |
| **No free drugs provided** | 29,549 | 0.26 | 0.18 |
| **Free drugs provided** | 13,013 | 0.42 | 0.42 |

**Figure SF3: Histograms of the estimated propensity scores by treatment status; N = 42,562**


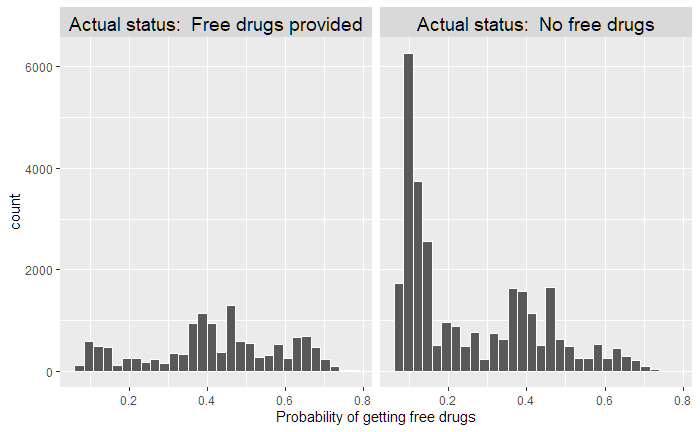


# Appendix 4 –Results from the Matching Procedure

Table S6 depicts the summary of balance achieved for matched dataset, post the propensity choice modeling. This is tabulated in comparison with the results obtained for the observational dataset.

**Table S6**

*Summary of balance achieved for matched dataset relative to the observational dataset*

|  | Unmatched Dataset (N = 42,562) | | | | | Matched Dataset; N = 23,242) | | | | |
| --- | --- | --- | --- | --- | --- | --- | --- | --- | --- | --- |
|  | Means Treated | Means Control | diff | Std. Mean Diff. | Var. Ratio | Means Treated | Means Control | diff | Std. Mean Diff. | Var. Ratio |
| distance | 0.42 | 0.26 | 0.17 | 0.96 | 1.01 | 0.39 | 0.39 | 0.00 | 0.00 | 1.00 |
| Age: 16-19 | 0.12 | 0.10 | 0.02 | 0.06 |  | 0.12 | 0.12 | 0.00 | 0.00 |  |
| Age: 20-45 | 0.62 | 0.59 | 0.04 | 0.08 |  | 0.62 | 0.62 | 0.00 | 0.00 |  |
| Age: 46-65 | 0.20 | 0.24 | -0.04 | -0.09 |  | 0.21 | 0.21 | 0.00 | 0.00 |  |
| Age: >=65 | 0.05 | 0.07 | -0.02 | -0.10 |  | 0.05 | 0.05 | 0.00 | 0.00 |  |
| Male | 0.56 | 0.58 | -0.02 | -0.04 |  | 0.56 | 0.56 | 0.00 | 0.00 |  |
| Xpert Testing | 0.33 | 0.15 | 0.18 | 0.38 |  | 0.26 | 0.26 | 0.00 | 0.00 |  |
| Extra Pulmonary | 0.32 | 0.31 | 0.02 | 0.04 |  | 0.33 | 0.33 | 0.00 | 0.00 |  |
| Ahmedabad | 0.30 | 0.13 | 0.17 | 0.38 |  | 0.26 | 0.25 | 0.00 | 0.00 |  |
| Bhopal | 0.13 | 0.08 | 0.05 | 0.15 |  | 0.13 | 0.12 | 0.01 | 0.03 |  |
| Darbhanga | 0.04 | 0.14 | -0.10 | -0.51 |  | 0.04 | 0.04 | 0.00 | 0.00 |  |
| Delhi | 0.14 | 0.42 | -0.28 | -0.80 |  | 0.16 | 0.16 | 0.00 | 0.00 |  |
| Gurgaon | 0.04 | 0.06 | -0.02 | -0.07 |  | 0.04 | 0.04 | 0.00 | 0.00 |  |
| Indore | 0.10 | 0.04 | 0.06 | 0.20 |  | 0.09 | 0.09 | 0.00 | -0.01 |  |
| Surat | 0.25 | 0.14 | 0.11 | 0.25 |  | 0.28 | 0.29 | -0.01 | -0.02 |  |

# Appendix 5 –Full Model Results; OLS Model; Matched Dataset

**Table S7**

*Results from the full OLS model on matched dataset; N = 23,242*

| Dependent Variable = Successful follow ups | |
| --- | --- |
| Constant | 5.037*** |
| Free drugs | 2.522*** |
| Xpert testing | 0.735*** |
| Age: 20-45 | -0.401*** |
| Age: 46-65 | -0.529*** |
| Age: > =65 | -1.435*** |
| Male | -0.298*** |
| Extrapulmonary | 1.076*** |
| Diag Qtr 2019 Q2 | 3.060*** |
| Diag Qtr 2019 Q3 | 6.024*** |
| Diag Qtr 2019 Q4 | 8.191*** |
| Diag Qtr 2020 Q1 | 9.274*** |
| Observations | 23,242 |
| R | 0.512 |
| Adjusted R | 0.509 |
| Residual Std. Error | 7.178 (df = 23087) |
| F Statistic | 157.568 (df = 154; 23087) |
| Note: a) 95% C.I. based on robust standard errors; b) Model was fitted on the matched dataset; c) *p<0.1; **p<0.05; ***p<0.01; d) TC fixed effects are excluded from the output; a total of 144 TC groups were fitted | |

# Appendix 6 –Full Model Results; Logistic Model; Matched Dataset

**Table S8**

*Logistic Regression; Impact of free drugs on Treatment Outcomes; N = 23,242*

| Dependent Variable: Binary Treatment Outcome (Successful=1, Unsuccessful = 0) | | |
| --- | --- | --- |
|  | **Coefficient** | **Odds Ratio** |
| (Intercept) | 16.89*** |  |
| Free drugs | 0.37*** | 1.45 |
| Xpert testing | -0.04 | 0.96 |
| Age: 20-45 | -0.25** | 0.78 |
| Age: 46-65 | -1.00*** | 0.37 |
| Age: > =65 | -1.67*** | 0.19 |
| Male | -0.13* | 0.88 |
| Extrapulmonary | 0.27*** | 1.31 |
| Diag Qtr 2019 Q2 | 0.101 | 1.11 |
| Diag Qtr 2019 Q3 | -0.073 | 0.93 |
| Diag Qtr 2019 Q4 | -0.089 | 0.91 |
| Diag Qtr 2020 Q1 | -0.123 | 0.88 |
|  | | |
| Observations | 23,242 |  |
| Log Likelihood | -4,690.780 |  |
| Akaike Inf. Crit. | 9,691.570 |  |
| ***Note****: a) 95% C.I. based on robust standard errors; b) Model was fitted on the matched dataset; c) *p<0.1; **p<0.05; ***p<0.01; d) TC fixed effects are excluded from the output; a total of 144 TC groups were fitted* | | |

# Appendix 7 –Sensitivity Analysis; Results from Simple OLS & Logistic Models; Matched and Unmatched (observational) datasets

**Table S9**

*Sensitivity analysis for OLS regression modelling the impact of free drugs on number of follow-ups*

|  | Coefficient | 95% C.I. | | Number of observations |
| --- | --- | --- | --- | --- |
| Base Model (Observational) | 2.531*** | 2.363 | 2.698 | 42,562 |
| Base Model (Matched) | 2.522*** | 2.325 | 2.719 | 23,242 |
| Base Model (Matched on TC) | 2.558*** | 2.351 | 2.764 | 19,436 |
| Only Ahmedabad | 3.452*** | 2.975 | 3.93 | 5,924 |
| Only Surat | 3.092*** | 2.676 | 3.509 | 6,652 |
| Only Delhi | 2.320*** | 1.908 | 2.731 | 3,614 |
| Only Gurgaon | 4.898*** | 4.018 | 5.778 | 1,028 |
| Only Indore | 1.839*** | 1.494 | 2.185 | 2,019 |
| Only Bhopal | 0.754*** | 0.405 | 1.102 | 2,995 |
| Only Darbhanga | 0.670** | 0.156 | 1.184 | 1,010 |
| Removing lost to follow ups (Observational) | 2.422*** | 2.254 | 2.59 | 40,965 |
| Including “excluded” outcomes (Observational) | 3.155*** | 2.985 | 3.324 | 44,293 |
| Including “excluded” + “early” outcomes (Observational) | 3.298*** | 3.126 | 3.470 | 45,072 |
| Patient group-pre Covid lockdown (Observational) | 2.098*** | 1.870 | 2.326 | 19,197 |
| Patient group-post Covid lockdown (Observational) | 2.747*** | 2.529 | 2.965 | 23,365 |
| Removing lost to follow ups (Matched) | 2.428*** | 2.23 | 2.626 | 22,622 |
| Removing lost to follow ups (Matched on TC) | 2.454*** | 2.246 | 2.661 | 18,935 |
| ***Note****: a) All models control for all available potential confounders, and include fixed effects for diagnosing quarter and treatment coordinator; b) 95% C.I. based on robust standard errors; c) LTFU refers to patient who were lost to follow-up, d) All district wise models are fitted on the matched dataset; e) Matched on TC refers to a matched dataset which was created by matching on treatment coordinator, and not on district. This alternative matching specification ensures an equal share of patients receiving free drugs and not receiving free drugs and results in 19,436 observations or 9718 matching pairs; f) *p<0.1; **p<0.05; ***p<0.01; g) Excluded outcomes refer to “not evaluated”, and “pending” outcomes, which were excluded in the base analysis – These were considered as unsuccessful for this sensitivity analyses; h) Early outcomes refer to outcomes which were declared within 30 days of diagnosing date, and were excluded from the base analysis; i) Patient groups pre & post lockdown consists of patients who had their outcomes declared before or after 25^th^ March 2020, thus identifying patients who weren’t or were under treatment during the nation wide COVID lockdown.* | | | | |

**Table S10**

*Sensitivity analysis for logistic regression, modelling the impact of free drugs on treatment outcomes*

|  | Free drugs | | | N observations |
| --- | --- | --- | --- | --- |
|  | Coeff | 95% C.I. | |  |
| Base Model (Observational) | 1.496*** | 1.353 | 1.654 | 42562 |
| Base Model (Matched) | 1.452*** | 1.288 | 1.637 | 23242 |
| Base Model (Matched on TC) | 1.42*** | 1.249 | 1.613 | 19436 |
| Only Ahmedabad | 1.244** | 0.950 | 1.631 | 5924 |
| Only Surat | 1.126 | 0.878 | 1.444 | 6652 |
| Only Delhi | 2.148*** | 1.563 | 2.951 | 3614 |
| Only Gurgaon | 1.869** | 1.060 | 3.297 | 1028 |
| Only Indore | 1.173 | 0.700 | 1.967 | 2019 |
| Only Bhopal | 1.407*** | 1.107 | 1.787 | 2995 |
| Only Darbhanga | 1.815*** | 1.030 | 3.197 | 1010 |
| Removing lost to follow ups (Observational) | 1.282*** | 1.121 | 1.467 | 40965 |
| Including “excluded” outcomes (Observational) | 1.845*** | 1.702 | 2.000 | 44,293 |
| Including “excluded” + “early” outcomes (Observational) | 1.902*** | 1.763 | 2.052 | 45,072 |
| Removing lost to follow ups (Matched) | 1.245** | 1.062 | 1.460 | 22622 |
| Removing lost to follow ups (Matched on TC) | 1.171* | 0.991 | 1.384 | 18935 |
| ***Note****: a) All models control for all potential confounders, and include fixed effects for diagnosing quarter and treatment coordinator; b) 95% C.I. based on robust standard errors; c) LTFU refers to patient who were lost to follow-up, d) All district wise models are fitted on the matched dataset; e) Matched on TC refers to a matched dataset which was created by matching on treatment coordinator, and not on district. This alternative matching specification ensures an equal share of patients receiving free drugs and not receiving free drugs and results in 19,436 observations or 9718 matching pairs; f) *p<0.1; **p<0.05; ***p<0.01* *g) Excluded outcomes refer to “not evaluated”, and “pending” outcomes, which were excluded in the base analysis – These were considered as unsuccessful for this sensitivity analyses; h) Early outcomes refer to outcomes which were declared within 30 days of diagnosing date, and were excluded from the base analysis; ; i) Patient groups pre & post lockdown consists of patients who had their outcomes declared before or after 25^th^ March 2020, thus identifying patients who weren’t or were under treatment during the nationwide COVID lockdown.* | | | | |

# Appendix 8– Definition of treatment outcomes

The definition of treatment outcomes considered in the study are provided below [7].

**Table S11**

*Definitions of treatment outcomes for drug susceptible TB patients*

| **Treatment outcome** | **Definition** | **Study outcome** | **Considered for the study** |
| --- | --- | --- | --- |
| Cured | Microbiologically confirmed TB patients at the beginning of treatment who was smear or culture negative at the end of the complete treatment | Successful | Yes |
| Treatment complete | Completed treatment without evidence of failure or clinical deterioration but with no record that the smear or culture results of biological specimen in the last month of treatment was negative |  |  |
| Lost to follow up | Treatment was interrupted for one consecutive month or more | Unsuccessful |  |
| Died | Died during the course of anti-TB treatment |  |  |
| Treatment failure | Biological specimen is positive by smear or culture at end of treatment |  |  |
| Not evaluated | Patients for whom no treatment outcome is assigned; also includes former transfer outs | Other outcomes (not considered) | No |
| Treatment regimen changed | A TB patient who is on first line regimen and has been diagnosed as having DR TB and switched to drug resistant TB regimen prior to being declared as failed |  |  |
| Wrongly diagnosed | A patient who is wrongly diagnosed of TB |  |  |
| Transferred | A patient who has transferred to another facility or state, prior to the outcome being declared |  |  |

# Appendix 9– Patients availing free drugs during different quarters

**Table S12**

*Share of patients availing free drugs during different time periods; N = 42,562*

| **Diagnosing quarter** | **private drugs** | **free drugs** | **Share of patients availing free drugs** |
| --- | --- | --- | --- |
| 2019 Q1 | 5,068 | 1,529 | 23.18% |
| 2019 Q2 | 7,133 | 2,405 | 25.21% |
| 2019 Q3 | 6,153 | 2,718 | 30.64% |
| 2019 Q4 | 5,462 | 2,852 | 34.30% |
| 2020 Q1 | 5,733 | 3,509 | 37.97% |

# References

1. Seo, Songwon. A Review and Comparison of Methods for Detecting Outliers in Univariate Data Setz. Master’s thesis. Univerity of Pittsburgh; 2006.

2. Walfish S. A review of statistical outlier methods. Pharmaceutical technology. 2006;30:82.

3. Iglewicz B, Hoaglin DC. How to detect and handle outliers. Milwaukee, Wis: ASQC Quality Press; 1993.

4. Rosenbaum PR, Rubin DB. The central role of the propensity score in observational studies for causal effects. Biometrika. 1983;70:41–55.

5. Winkelmayer WC, Kurth T. Propensity scores: help or hype? Nephrology Dialysis Transplantation. 2004;19:1671–3.

6. Heinrich C, Maffioli A, Vázquez G. A Primer for Applying Propensity-Score Matching. SPD Working Paper. Inter-American Development Bank, Office of Strategic Planning and Development Effectiveness (SPD); 2010.

7. Central TB Division. Technical and Operational Guidelines for TB Control in India [Ebook]. In: Technical and Operational Guidelines for TB Control in India. New Delhi, India: Ministry of Health & Family Welfare; 2016. p. Chapter 4.
